# Management von medikamentenassoziierten Kiefernekrosen – Ergebnisse einer Literaturanalyse neuester Studien im Vergleich zu bewährten Strategien

**DOI:** 10.1007/s00106-021-01130-0

**Published:** 2022-01-20

**Authors:** Matthias Tröltzsch, Markus Tröltzsch, Christoph Pautke, Sven Otto

**Affiliations:** 1 Zentrum für Zahn- Mund- und Kieferheilkunde Ansbach, Ansbach, Deutschland; 2 Klinik und Poliklinik für Mund‑, Kiefer- und Gesichtschirurgie, Ludwig-Maximilians-Universität München, Lindwurmstr. 2A, 80337 München, Deutschland; 3 Medizin und Ästhetik, München, Deutschland; 4 Klinik und Poliklinik für Mund‑, Kiefer- und Gesichtschirurgie, Martin-Luther-Universität Halle-Wittenberg, Halle, Deutschland

**Keywords:** MRONJ, Kieferosteonekrose, Bisphosphonate, Denosumab, Fluoreszenzgesteuerte Chirurgie, Onkologie, MRONJ, Osteonecrosis of the jaw, Bisphosphonates, Denosumab, Fluorescence-guided surgery, Oncology

## Abstract

**Hintergrund:**

Antiresorptiva gehören weltweit zu den am häufigsten applizierten Arzneimitteln. Ihr Haupteinsatzbereich liegt in der Osteologie und Onkologie. Trotz allgemein guter Verträglichkeit treten bei Patienten unter Therapie unerwünschte Arzneimittelwirkungen (UAW) auf. Eine spezifische UAW im Bereich der Kiefer ist die sog. medikamentenassoziierte Osteonekrose („medication-related osteonecrosis of the jaw“, MRONJ) der Kiefer.

**Ziel der Arbeit:**

Diese Arbeit stellt neuesten Entwicklungen in Ätiologie, Diagnostik und Therapie der MRONJ im Vergleich zu bereits bestehenden Erkenntnissen zusammen.

**Methodik:**

Es wurde eine systematische Literaturübersicht der Jahre 2016–2021 zu diesem Thema durchgeführt. Prospektive Therapiestudien, Diagnostikstudien mit Vergleichsgruppe und innovative Studien zur Pathogenese der MRONJ wurden eingeschlossen und nach den MINORS-Kriterien („methodological index for non-randomized studies“) bewertet.

**Ergebnisse und Diskussion:**

Die MRONJ tritt bei ca. 2–12 % der Patienten, die aus onkologischer Indikation mit Antiresorptiva behandelt werden, auf (osteologische Indikation ca. 0,1–1 %). Die Therapie der MRONJ sollte frühzeitig und operativ erfolgen. Die Heilungsrate ist bei einem operativen Therapieansatz mit über 85 % sehr gut.

## Abstract (en)

**Background:**

Antiresorptive agents are some of the most frequently used drugs worldwide, with indications in osteology and oncology. They are generally well tolerated and display a favorable safety profile. A potentially severe unwanted side effect is medication-related osteonecrosis of the jaw (MRONJ).

**Purpose of this review:**

This review summarizes the latest developments in etiology, diagnosis, and treatment of MRONJ, and compares new insights with established algorithms.

**Methods:**

A systematic review of relevant studies exploring diagnostic methods, prospective management trials, and innovative studies on the pathogenesis of MRONJ published between 2016 and 2021 was performed. The study quality was assessed using the MINORS (methodological index for non-randomized studies) rating score.

**Results and discussion:**

The prevalence of MRONJ in patients undergoing treatment with antiresorptive drugs for oncological reasons is remarkable (2–12%). MRONJ prevalence in patients receiving antiresorptive drugs for the treatment of osteoporosis is much lower (0.1–1%). MRONJ treatment should be initiated early and involve a surgical approach. MRONJ treatment is safe and predictable, with long-term success rates of more than 85%.

Unter Antiresorptiva werden vor allem die Medikamentengruppen der Bisphosphonate (BP) und der Antikörper Denosumab (DNO) verstanden. Es handelt sich um hochwirksame Medikamente, die im Wesentlichen durch Hemmung der Osteoklastenaktivität zu einer Verminderung des physiologischen „bone remodelling“ führen [37]. Die Haupteinsatzbereiche der Antiresorptiva liegen in der Osteologie und Onkologie. Sie werden weltweit millionenfach täglich eingesetzt und weisen grundsätzlich ein hohes Sicherheitsprofil auf [41]. Die klinischen Haupteffekte der Antiresorptiva sind die signifikante Reduktion osteoporotisch oder neoplastisch induzierter pathologischer Frakturen (mit Reduktion der Mortalität) [3], die Reduktion von tumorassoziierten Knochenschmerzen und die Senkung des Blutkalziumspiegels insbesondere bei osteolytischen Prozessen [26].

## Pharmakologische Aspekte

Bei BP werden stickstofffreie (z. B. Clodronat) und stickstoffhaltige (z. B. Zoledronat) Pharmaka unterschieden, die zwar unterschiedliche Wirkungen auf den Zellstoffwechsel haben, jedoch in der Endstrecke dieselbe Wirkung erzielen [39]. Sie können kovalent an das Hydroxylapatit des Knochens mit hoher Organspezifität binden [37]. Sie haben eine lange Halbwertszeit (mehrere Jahre) [11]. Eine Übersicht der aktuell am häufigsten verwendeten Bisphosphonate ist Tab. 1 zu entnehmen.

**Table 1:** 

Wirkstoff	Applikationsart	Indikation
Alendronsäure	Oral	Osteoporose
Risedronsäure	Oral	Osteoporose
Ibandronsäure	Oral und intravenös	Osteoporose/Onkologie
Zoledronsäure	Intravenös	Osteoporose/Onkologie

Als weiteres antiresorptiv wirksames Pharmakon steht der monoklonale Antikörper DNO zur Verfügung (subkutane Applikation; 60 mg halbjährlich oder 120 mg monatlich). Der molekulare Wirkmechanismus basiert auf der zielgerichteten Hemmung des sog. RANK-Liganden (Rezeptoraktivator des nukleären Faktors Kappa B – Ligand), der von Osteoblasten sezerniert werden kann und die Osteoklastenaktivität stimuliert [45]. Somit zeichnet sich Denosumab durch eine hohe Zellspezifität aus. Aufgrund der im Vergleich zu Bisphosphonaten grundsätzlich unterschiedlichen Pharmakokinetik beträgt die Plasmahalbwertszeit von Denosumab nur ca. 26 Tage [1].

## Unerwünschte Arzneimittelwirkungen von Antiresorptiva

Gemessen an der Häufigkeit ihres Einsatzes und ihrem großem Nutzen sind Antiresorptiva sehr sichere Arzneimittel [41]. Selten können bei oraler Applikation gastrointestinale unerwünschte Arzneimittelwirkungen (UAW) oder bei intravenöser (BP) oder subkutaner (DNO) Applikation grippeähnliche Symptome auftreten [41]. Eine für die Kopf-Hals-Medizin bedeutsame UAW der Antiresorptiva ist das Auftreten chronischer Wundheilungsstörungen der Kiefer mit Absterben von Kieferbereichen (Knochennekrose) bei regelmäßiger Applikation, insbesondere in hoher Dosis [14]. Da auch andere Medikamente (neben Antiresorptiva) mit der Entstehung von Nekrosen der Kieferknochen assoziiert werden, wird das Krankheitsbild in der aktuellen Literatur oft als sog. medikamentenassoziierte Kiefernekrose („medication-related osteonecrosis of the jaw“, MRONJ) bezeichnet. Dabei handelt es sich vor allem um Angiogenesehemmer (z. B. Sunitinib, Imatinib, Bevacizumab), mTOR-Inhibitoren und BRAF-Inhibitoren [19], die fast nur in onkologischen Therapieregimen zum Einsatz kommen.

## Krankheitsbild der medikamentenassoziierten Kiefernekrose

**Figure 1:**
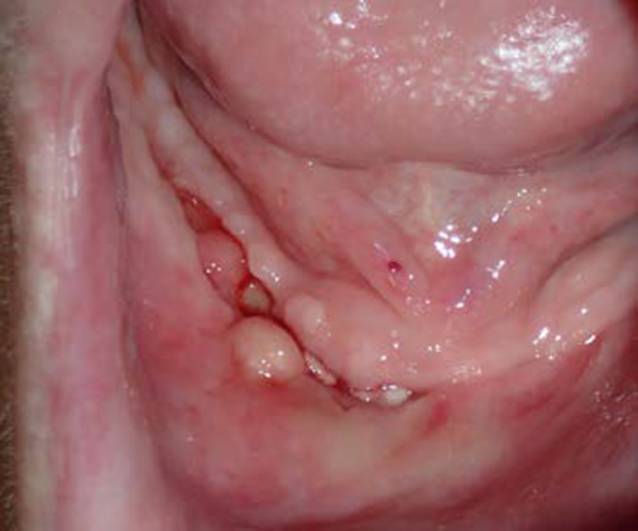

Klinisch zeigen sich Dehiszenzen der Mundschleimhaut mit entweder sondierbarem oder flächig freiliegendem Knochen (Abb. 1) mit oder ohne lokale oder/und systemische Entzündungszeichen. Neurologische Beeinträchtigungen der Funktion des N. alveolaris inferior mit Hypästhesien im Bereich der Unterlippe, Sequesterbildungen im Bereich des Unterkieferknochens bis hin zur pathologischen Fraktur sowie eine Beteiligung der Nasennebenhöhlen bei Erscheinungsformen der MRONJ im Oberkiefer können als Komplikationen auftreten [38]. Aktuell sind vier Stadien der MRONJ definiert (Tab. 2).

**Table 2:** 

Stadium 0	Nekrotischer Knochen bei geschlossener Mundschleimhaut wird vermutet (radiologisch oder klinisch)
Stadium I	Freiliegender Knochen ohne klinische Beschwerden
Stadium II	Freiliegender Kieferknochen mit klinischen Beschwerden (z. B. Infektion)
Stadium III	Freiliegender Kieferknochen mit zusätzlichen Komplikationen (Kieferfraktur, Sinusitis, oroantrale oder extraorale Fisteln)

Der rasch voranschreitende wissenschaftliche Fortschritt in den Bereichen Pathogenese, Diagnostik und Therapie dieses speziellen Krankheitsbilds erschwert es praktisch tätigen Ärzten, die für den klinischen Alltag wichtigen Informationen zu filtern. Ziel der vorliegenden Literaturübersicht ist die Zusammenfassung der aktuellsten Erkenntnisse zur den Themen Pathogenese, Diagnostik und Therapie von medikamentenassoziierten Kiefernekrosen und die Gegenüberstellung dieser Daten zu bereits bekannten Prinzipien.

## Methodik

Zur Gewinnung relevanter wissenschaftlicher Informationen in dieser Fragestellung wurde eine systematische Literaturrecherche entsprechend den PRISMA-Vorgaben [17] in folgenden englischsprachigen Datenbanken durchgeführt: PubMed, Web of Science, Google Scholar. Der Zeitraum der Literaturrecherche wurde auf die Jahre 2016–2021 begrenzt. Zur Formulierung einer geeigneten wissenschaftlichen Fragestellung im Hinblick auf die aktuellen Empfehlungen zur Therapie wurde das „PICO-Konzept“ genutzt: P = Patient:innen mit MRONJ, I = operative Therapie, C = konservative Therapie (wenn untersucht), O = Heilung (Suchworte „therapy“ AND „MRONJ“). Daraus wurde folgende Fragestellung abgeleitet: „Wie hoch ist die Erfolgsrate (O) operativer Therapieansätze (I) im Vergleich zu nichtoperativen Therapieansätzen (C) bei Patient:innen mit MRONJ (P)?“.

Da sich dieses Vorgehen nicht auf den Aspekt „Diagnostik der MRONJ“ und ausweiten ließ, wurde eine weitere Literatursuche mit den Begriffen „diagnostic methods“ AND „MRONJ“ durchgeführt. Die Qualität der eingeschlossenen Studien wurde mithilfe des „methodological index for non-randomized studies“ (MINORS) bewertet [42]. Weiterentwicklungen der Pathogenesetheorie der MRONJ wurden aus einer selektiven Literaturanalyse mit den Suchbegriffen „pathogenesis“ AND „MRONJ“ im gleichen Zeitraum entnommen. Die Ergebnisse der aktuellen Literaturanalyse wurden zur Hervorhebung von Neuerungen mit älteren Erkenntnissen verglichen. Präklinische Studien, Literaturübersichten und retrospektive Studien wurden von der systematischen Auswertung hinsichtlich Diagnostik und Therapie der MRONJ ausgeschlossen.

In die Literaturauswertung zur Diagnostik und Therapie der MRONJ wurden nur (prospektiv angelegte) Humanstudien eingeschlossen.

## Ergebnisse

Aufgrund der Änderung der Nomenklatur des Krankheitsbilds, das zuerst als bisphosphonatassoziierte („bisphosphonate-related“) Osteonekrose der Kiefer (BRONJ), später als antiresorptivaassoziierte („antiresorptive agent-related“) Osteonekrose der Kiefer (ARONJ) und nun seit 2014 als MRONJ bezeichnet wurde [38], gibt es keine Treffer bei Literaturrecherchen zu diesem Thema vor dem Jahr 2014.

Die Literaturanalyse zur PICO-Fragestellungen ergab insgesamt 70 Studien. Nach Anwendung der Ausschlusskriterien blieben noch 6 Arbeiten, die sich für eine Datenauswertung eigneten (Tab. 3; [5, 6, 8, 13, 15, 27]). Trotz umfassender wissenschaftlicher Anstrengungen zur Verbesserung der Therapiekonzepte der MRONJ existieren nur wenige prospektive Arbeiten. Allen Studien gemeinsam sind eine limitierte Fallzahl und das Fehlen einer Kontrollgruppe (im Sinne einer nicht vorhandenen Intervention oder einer parallel geführten Kohorte, bei der konservative Therapieregime angewandt wurden). Der Therapieerfolg wurde in allen Studien als die komplette Abheilung der MRONJ-Läsion definiert. Für therapierefraktäre MRONJ-Läsionen wurde eine Reduktion des MRONJ-Stadiums als Therapieerfolg angesehen.

**Table 3:** 

Autor, Jahr	Größe der Studienkohorte	Dauer der Nachuntersuchung	Eingeschlossene MRONJ-Stadien	Kontrollgruppe	Art der Therapie	Therapieerfolgsrate	Verbesserung des MRONJ-Stadiums	MINORS-Score
Klingelhöffer et al. 2016 [12]	40	55 Wochen	I–III	Nein	Operative Therapie	27,6 %	83 %	16/24
Otto et al. 2016 [26]	54	56 Wochen	I–III	Nein	FGS	86 %	> 90 %	18/24
Mauceri et al. 2017 [14]	10	52 Wochen	I–II	Nein	Lasertherapie und „platelet-rich plasma“	30 %	57 %	12/24
Giudice et al. 2018 [5]	36 (insgesamt 39 MRONJ-Läsionen)	52 Wochen	I–III	Ja	AFGS vs. operative Therapie	AFGS: 15/19 (78 %)	Nicht angegeben	20/24
						Operative Therapie: 14/20 (70 %)		
Hallmer et al. 2018 [7]	55	8 Wochen	I–III	Nein	Operative Therapie	80–92 % (je nach Verfahren)	Nicht angegeben	18/24
Giudice et al. 2020 [4]	129	15 Wochen	I–II	Nein	Operative Therapie	> 90 %	Nicht angegeben	20/24

Nur in einer Studie wurde ein wenig invasiver Therapieansatz zur Therapie der MRONJ angewandt [15]. Diese wies allerdings eine sehr geringe Fallzahl auf, und ein Abheilen der MRONJ-Läsionen wurde nur bei 3/10 Fällen erreicht. Alle anderen Arbeiten untersuchten die Erfolgsraten operativer Verfahren von MRONJ-Läsionen (das operative Vorgehen wird in diesem Manuskript anhand eines Fallbeispiels beschrieben) [5, 6, 8, 13, 27, 34]. Mit Ausnahme einer Publikation [13] wurden durchweg hohe und dauerhafte Heilungschancen durch operative therapeutische Ansätze der MRONJ berichtet. Der MINORS-Score der begutachteten Therapiestudien lag zwischen 50 % und knapp über 80 %. Die Qualität der Datenlage ist somit als moderat zu bewerten.

Die Literaturrecherche hinsichtlich der neuen Aspekte zur Diagnostik der MRONJ ergab 271 Treffer. Nach Anwendung der Ausschlusskriterien waren noch 7 Studien zur Auswertung geeignet (Tab. 4; [7, 9, 18, 21, 22, 40, 44]). Die meisten Studien zur Diagnostik untersuchten die Ergebnisqualität unterschiedlicher MRT-Verfahren und nuklearmedizinischer Diagnostik in der Differenzierung zwischen MRONJ und gesunden Knochenarealen oder von MRONJ im Unterschied zu anderen entzündlichen Erkrankungen der Kieferknochen. Die Ergebnisse der entsprechenden Studien zeigen eine hohe Genauigkeit für MRT-basierte Analysen hinsichtlich der Erkennung von MRONJ und der Differenzierung zu gesunden Knochenarealen. Nuklearmedizinische Verfahren diagnostizieren MRONJ-Läsionen ebenfalls sehr genau und eignen sich besonders zur Unterscheidung unterschiedlicher ossärer Pathologien der Kiefer. Die durchschnittliche Qualität der Studien lag nach Auswertung des MINORS-Score wie bei den Therapiestudien zwischen 50 % und ca. 80 % und war somit ebenfalls nur moderat.

**Table 4:** 

Autor, Jahr	Größe der Studienkohorte	Untersuchtes diagnostisches Verfahren	Vergleichsgruppe	Untersuchter Parameter	Ergebnisse	MINORS-Score
Guo et al. 2016 [6]	40	OPT vs. CT	Keine	Radiologische Kriterien der MRONJ	CT bei geringen MRONJ-Stadien genauer als OPT	20/24
Ogura et al. 2019 [20]	13 (9 MRONJ, 4 Osteomyelitis)	SPECT/CT	MRONJ vs. Osteomyelitis	„Standardized bone uptake“ (SUV)	SUV bei Osteomyelitis signifikant höher als bei MRONJ	12/24
Ogura et al. 2019 [21]	7 (3 Osteoradionekrose, 3 MRONJ, 1 RA)	SPECT/CT	MRONJ vs. Osteoradionekrose	„Standardized bone uptake“ (SUV)	SUV bei MRONJ höher als bei Osteoradionekrose	12/24
Huber et al. 2019 [8]	19	DVT vs. Ultrashort-Echo-Time-MRT	MRONJ vs. nicht betroffene Kieferbereiche	Grauwerte bzw. Signalintensität pro ROI/VOI	Nichtunterlegenheit des MRT gezeigt	16/24
Toshima et al. 2020 [43]	44 (7 Osteomyelitis, 8 Osteoradionekrose, 29 MRONJ)	SPECT/CT	MRONJ vs. Osteomyelitis vs. Osteoradionekrose	„Standardized bone uptake“ (SUV)	SUV bei Osteomyelitis signifikant höher als bei MRONJ, am geringsten bei Osteoradionekrose	18/24
Schumann et al. 2021 [39]	20	MRT mit Kontrastmittel (KM)	MRONJ vs. nicht betroffene Knochenanteile	KM-Dynamik	Untersuchte Parameter in MRONJ signifikant höher als in nicht betroffenen Knochenarealen	20/24
Muraoka et al. 2021 [17]	38	MRT	Kontrollgruppe ohne MRONJ (10 Probanden)	Diffusionskoeffizient	Deutliche Unterschiede zwischen verschiedenen MRONJ-Stadien	20/24

Die Literatursuche zur Pathogenese der MRONJ ergab 186 Treffer, von denen schließlich 8 Arbeiten berücksichtigt wurden [10, 12, 16, 20, 31, 32, 43, 46]. Die bereits formulierte Pathogenesetheorie, dass chronische Entzündungen ursächlich mit der Entstehung von MRONJ verbunden sind, wird in allen Studien bestätigt. Dentoalveoläre chirurgische Maßnahmen werden klinisch nach wie vor mit der Entwicklung einer MRONJ in Verbindung gebracht [16, 46]. Allerdings verdichten sich die Hinweise, dass nekrotische Knochenareale (also die eigentliche MRONJ) zum Zeitpunkt der chirurgischen Intervention bereits vorgelegen und sich nicht erst danach entwickelt haben [20, 32, 43].

## Diskussion

Ziel der vorliegenden Literaturübersicht war die Zusammenfassung von aktuellen Erkenntnissen in Pathogenese, Diagnostik und Therapie und die Herausarbeitung von Neuerungen.

Das Krankheitsbild der MRONJ ist in der Kopf-Hals-Medizin relativ häufig und auch in der Hals-Nasen-Ohren-Heilkunde durch MRONJ-Fälle am äußeren Gehörgang bekannt [2]. Die Prävalenz der Erkrankung in Patientenkollektiven, die aufgrund osteologischer Indikation mit Antiresorptiva behandelt werden, beträgt je nach Studie zwischen 0,1 % und 1 %. In onkologischen Patientenkollektiven liegt diese Rate mit 2–12 % wesentlich höher [26]. Diese UAW wird bei Patienten, die unter Therapie mit Denosumab stehen, ebenfalls in ähnlicher Häufigkeit beobachtet [45].

### Pathogenese der MRONJ

Alle im Rahmen dieses systematischen Reviews ausgewerteten Studien zur Pathogenese bestätigen die bereits anerkannte Theorie, dass chronisch entzündliche Prozesse im Bereich der Kiefer die Ursache der MRONJ darstellen [23–25, 30]. Allen Antiresorptiva gemeinsam ist die negative Beeinflussung der osteoklastischen Aktivität. Dabei spielt vor allem der niedrige pH-Wert in MRONJ-Läsionen eine große Rolle [12, 25, 30]. Durch diese Azidifizierung werden BP aus ihrer Bindung an Hydroxylapatit bis zum Erreichen toxischer lokaler Werte herausgelöst [12]. Dies beeinflusst neben den Osteoklasten auch noch Endothelzellen, Epithelzellen und Immunzellen [33]. In der Endstrecke behindern diese Effekte eine problemlose Wundheilung, und MRONJ-Läsionen können entstehen. Daraus ergibt sich die klinische Beobachtung, dass nach operativen Eingriffen in der Mundhöhle im entsprechenden Risikokollektiv eine MRONJ getriggert werden kann [29]. Durch groß angelegte, multizentrische Studien [26] konnten Faktoren identifiziert werden, die die Wahrscheinlichkeit des Auftretens einer derartigen Nekrose erhöhen können (Tab. 5). Die exakte Pathogenesetheorie der Kiefernekrose nach Applikation von Denosumab ist noch nicht vollständig aufgeklärt. Neueste Studienergebnisse deuten darauf hin, dass es doch Unterschiede im klinischen Verlauf zwischen ARONJ als Folge von BP-Therapie oder DNO-Therapie geben könnte [31]. Das Risiko für das Auftreten einer MRONJ scheint bei Patientinnen und Patienten unter Therapie mit DNO wohl frühzeitig höher zu liegen als bei BP-Therapie [31]. Ob ein erhöhtes Risiko für das Auftreten einer MRONJ beim Wechsel von BP auf DNO vorliegt, ist noch nicht abschließend geklärt [10, 31].

**Table 5:** 

Niedrigeres Risiko	Erhöhtes Risiko
Therapie mit Bisphosphonaten oder Denosumab in niedriger Dosis (zumeist osteologische Indikation)	Therapie mit Bisphosphonaten oder Denosumab in erhöhter Dosis (zumeist onkologische Indikation)
Kürzere Therapiedauer (zumeist weniger als 3 Jahre)	Längere Therapiedauer (zumeist mehr als 3 Jahre)
Nichtraucher	Raucher
Keine immunsuppressive Vorerkrankung (z. B. Diabetes mellitus)	Immunsuppression (z. B. Diabetes mellitus, immunsuppressive Medikation)
Keine Komedikation mit Steroiden, mTOR-Inhibitoren, Tyrosinkinaseinhibitoren oder Angiogenesehemmern	Komedikation mit Steroiden, mTOR-Inhibitoren, Tyrosinkinaseinhibitoren oder Angiogenesehemmern

Aus der Pathogenesetheorie wird klar, dass die effektive Vermeidung entzündlicher Vorgänge im Bereich der Kieferknochen das Risiko für die Entstehung einer MRONJ senkt [4]. Zu den zentralen Aspekten der MRONJ-Prophylaxe gehören eine Vorstellung der Patient:innen bei einem spezialisierten Arzt (Zahnarzt, MKG-Chirurg) vor Beginn der Antiresorptivatherapie, um ein individuelles Risikoprofil erstellen zu lassen.

### Diagnostik und Therapie der MRONJ

Es existieren unterschiedliche Protokolle zur Therapie der MRONJ. Ein nichtoperativer Therapieansatz mit Antibiotikatherapie, antiseptischen Spülungen und Elimination der mutmaßlich auslösenden Faktoren der MRONJ kann für limitierte Krankheitsstadien versucht werden [35]. Allerdings scheint sich der Erfolg der nichtoperativen Therapie im Wesentlichen in einer Reduktion der Transformation der MRONJ in höhere Stadien zu äußern [36]. Eine komplette Ausheilung der MRONJ-Läsionen unter nichtoperativer Therapie ist nach aktuellem Kenntnisstand äußerst unwahrscheinlich [35]. Diese Meinung wird durch die Ergebnisse der aktuellen Literaturanalyse unterstützt. Alle ausgewerteten Studien zur Therapie der MRONJ befassten sich mit chirurgischen Therapieansätzen und berichteten über Erfolgsraten von bis zu 90 % (komplette Abheilung) bei überwiegend hoher Studienqualität. Die fluoreszenzgesteuerte MRONJ-Chirurgie stellte sich in zwei prospektiv angelegten Untersuchungen als besonders erfolgversprechend heraus [6, 27]. Neuere prospektive Studien zur nichtoperativen Therapie der MRONJ konnten nicht gefunden werden.

Die Literaturanalyse führte keine Studien zutage, in denen der Nutzen einer Pausierung der Antiresorptivamedikation („drug holiday“) vor einem operativen Vorgehen prospektiv untersucht wurde. In älteren, teilweise retrospektiv angelegten Arbeiten wurde eine „drug holiday“ empfohlen, um die immunmodulatorischen und möglicherweise weichgewebstoxischen Wirkungen zu minimieren [39]. Eine mehrwöchige Pausierung einer Denosumabtherapie führt aufgrund der kürzeren Halbwertszeit zu einer Erholung des „bone remodelling“, wodurch möglicherweise die Heilungsraten der Therapie erhöht werden können [39].

In der Diagnostik der MRONJ kommen nach wie vor konventionelle (zahn)ärztliche Röntgenverfahren (z. B. Orthopantomogramm), Schnittbildverfahren (DVT, CT, MRT) und nuklearmedizinische Verfahren zum Einsatz [9]. Die neuesten Studien untersuchen die Verfeinerung der diagnostischen Aussagekraft bereits bekannter Techniken [9, 18, 21, 40, 44].

Die Translation der wissenschaftlichen Erkenntnisse in den klinischen Alltag wird im Folgenden anhand einer Kasuistik beschrieben.

### Fallbeschreibung

Eine 64-jährige Patientin stellte sich auf Überweisung ihres behandelnden Gynäkologen aufgrund rezidivierender Schmerzen im Bereich des Unterkiefers vor. Anamnestisch lag ein ossär und viszeral metastasiertes Mammakarzinom vor. Das Therapieschema umfasste eine mehrjährige Therapie mit Antiresorptiva (zunächst für 4 Jahre Zoledronat 4 mg intravenös, 1 × monatlich). Vor 8 Monaten sei die Therapie von Zoledronat auf Denosumab (Xgeva 120 mg 1 × monatlich s.c.) umgestellt worden. Die ossäre Metastasierung sei in den vergangenen Jahren nicht progredient. Vor 4 Monaten sei im Bereich des Unterkiefers rechtsseitig ein parodontal zerstörter Zahn von ihrem Zahnarzt entfernt worden. Seither leide sie regelmäßig unter Schmerzen in dieser Region.

Klinisch zeigte sich eine chronische Wundheilungsstörung mit freiliegendem Knochen im Bereich des Unterkiefers rechtsseitig (Abb. 1), sodass die Diagnose einer MRONJ (Stadium I) gestellt wurde. Nach einer 3‑monatigen Denosumab-Pausierung wurde eine operative Therapie der MRONJ durchgeführt. Perioperativ ist grundsätzlich eine Antibiotikatherapie empfehlenswert. Diese wird bereits präoperativ begonnen und auch postoperativ für mindestens 10–14 Tage weitergeführt. Dieser Zeitraum kann je nach klinischem Befund auch verlängert werden. Penicilline mit breitem Spektrum und Zusatz von Beta-Laktamase-Inhibitoren haben sich für diese Indikation als besonders geeignet erwiesen.

**Figure 2:**
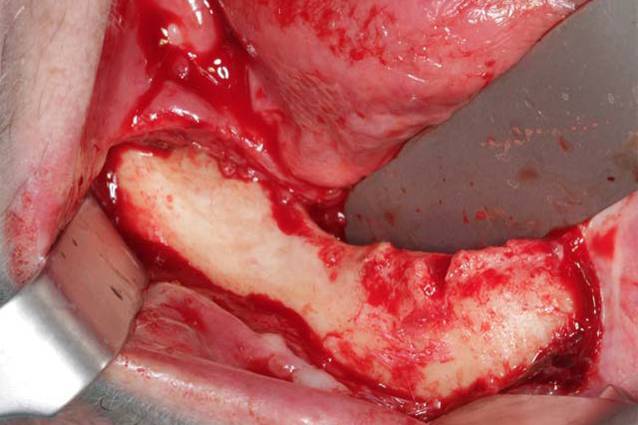

**Figure 3:**
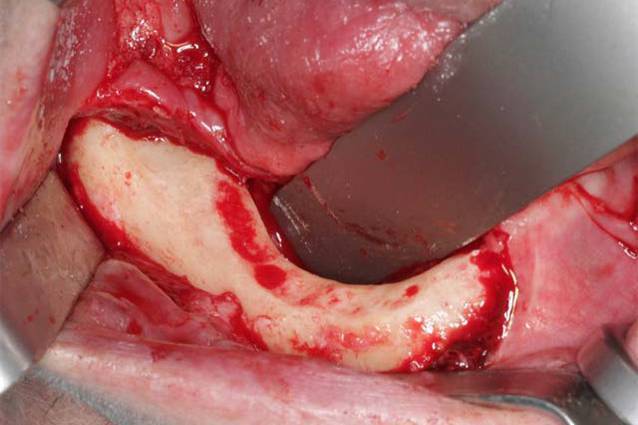

**Figure 4:**
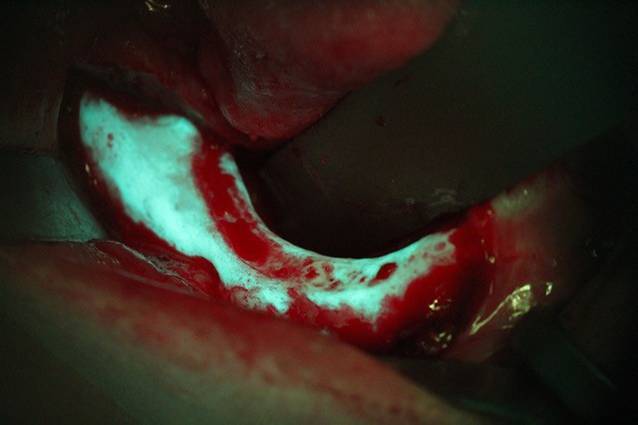

**Figure 5:**
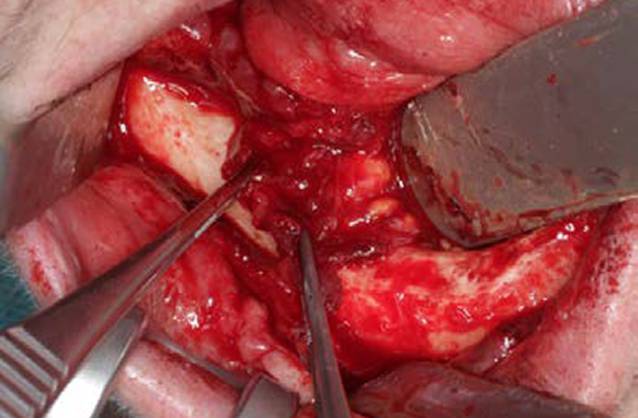

**Figure 6:**
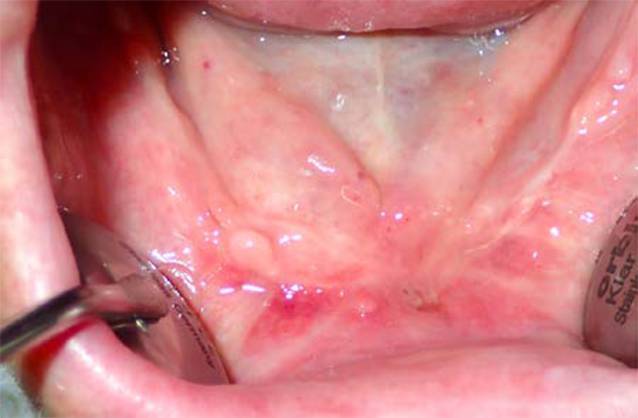

Die operative Therapie der MRONJ umfasst die Exploration im betroffenen Bereich und die Darstellung der gesamten Ausdehnung der Läsion (Abb. 2). Dann erfolgt die Resektion der nekrotischen Knochenareale (Abb. 3). Dies kann rein visuell oder fluoreszenzgesteuert durchgeführt werden (Abb. 4). Der entscheidende Vorteil der fluoreszenzgesteuerten MRONJ-Chirurgie besteht darin, dass durch Autofluoreszenz oder wirkstoffinduzierte Fluoreszenz vitale Knochenareale mit entsprechenden Lampen von nekrotischen Knochenarealen unterschieden werden können [27, 30, 34]. Dies ermöglicht eine zielgerichtete und standardisierte Therapie mit reduzierter Invasivität. Entscheidend für den Heilungsprozess ist ein möglichst dichter, spannungsfreier und wenn möglich mehrschichtiger Wundverschluss. In diesem Fall wurden als zusätzliche Schicht im Rahmen des Wundverschlusses Fasern des M. mylohyoideus präpariert, über den Alveolarkamm nach vestibulär mobilisiert und dort fixiert (Abb. 5). In 80–90 % der Fälle kann durch dieses Vorgehen eine primäre, vollständige und weitgehend komplikationslose Wundheilung erreicht werden ([28]; Abb. 6).

## Fazit für die Praxis

- Als Antiresorptiva werden im Wesentlichen die Medikamentengruppe der Bisphosphonate und der monoklonale Antikörper Denosumab verstanden.
- Die Halbwertszeit der Bisphosphonate beträgt Monate bis Jahre, während die des Denosumab 4–6 Wochen beträgt.
- Hauptwirkung der Antiresorptiva ist die Reduktion des physiologischen „bone remodelling“ durch Hemmung der Osteoklastentätigkeit.
- Haupteinsatzbereiche der Antiresorptiva sind die Osteoporose und die Therapie osteolytischer Metastasen.
- Eine unerwünschte Arzneimittelwirkung beider Medikamente ist die MRONJ.
- Die Prävalenz der Erkrankung bei onkologischer Dosierung beträgt ca. 2–12 %, bei osteologischer Dosierung beträgt ca. 0,1–1 %.
- Bei der MRONJ handelt es sich um Läsionen des Kieferknochens, die zumeist mit chronischen Wundheilungsstörungen, freiliegenden Knochenanteilen und unterschiedlich ausgeprägter Entzündungssymptomatik einhergehen können.
- Die Therapie der MRONJ sollte frühzeitig und chirurgisch erfolgen.
- Die Erfolgswahrscheinlichkeit bei einer frühzeitigen chirurgischen Behandlung liegt bei 80–90 % (vs. weniger als 20 % bei nichtoperativer Therapie).
